# Effect of Intracanal Glass-Ionomer Barrier Thickness on Microleakage in Coronal Part of Root in Endodontically Treated Teeth: an In Vitro Study

**DOI:** 10.30476/DENTJODS.2019.77830

**Published:** 2020-03

**Authors:** Armaghan Alikhani, Maryam Babaahmadi, Najme Etemadi

**Affiliations:** 1 Dept. of Operative Dentistry, School of Dentistry, Ahvaz University of Medical Sciences, Iran; 2 Dentist, School of Dentistry, Ahvaz University of Medical Sciences, Iran; 3 Resident of Operative Dentistry, School of Dentistry, Ahvaz University of Medical Sciences, Iran

**Keywords:** Endodontically treated teeth, Glass-ionomer, Sealing

## Abstract

**Statement of the Problem:**

The most common cause of endodontic treatment failures is improper coronal sealing. Therefore, besides to proper root sealing, coronal sealing which is supported by a proper restoration has a major role in endodontic treatment success, and coronal microleakage should be considered as an etiologic factor in endodontic treatment failure. Glass-ionomer (GI) has been proposed as a coronal barrier for microleakage after endodontic treatment.

**Purpose:**

This study aimed to evaluate the coronal microleakage in GI-obturated root canals in endodontically treated teeth using different thicknesses of GI.

**Materials and Method:**

In this in vitro study, forty-five single-rooted extracted human teeth with single canals were collected and disinfected with 0.5% chloramine solution. After endodontic treatment, teeth were divided into 3 groups. In the group 1 to 3, 1 to 3mm of gutta-percha was removed and GI was replaced at 1-, 2- and 3-mm thicknesses respectively. Then subgroups were placed in methylene blue dye and the microleakage was assessed using dye penetration.

**Results:**

The mean dye penetration in groups 1, 2 and 3 were 5.1, 3.7 and 2.9, respectively, with statistically significant differences. Group 1 exhibited the highest amount of dye penetration while group 3 showed the least one. Moreover, a significant difference between groups 1 and 2 (*p*= 0.002) and a non-significant difference between groups 2 and 3 (*p*= 0.098) was detected in mean dye penetration.

**Conclusion:**

Thicker layers of GI might decrease the coronal microleakage. GI at 3-mm thickness resulted in the best protective effect on coronal microleakage in endodontically treated teeth, though further studies are needed to confirm these results.

## Introduction

Root canal contamination is usually prevented by a crown restoration. Coronal microleakage is considered as a major factor related to endodontic treatment failure. Currently, more attention is paid to the quality of the final restoration [ 1 ]. One of the most important techniques to prevent penetration of microorganisms and saliva into the root canal is the sealing of cavity access [ 2 ]. Moreover, researchers have shown that endodontic treatment of teeth exhibits a higher failure rate when coronal sealing is not appropriate [ 3 ]. The most common coronal sealers include mineral trioxide aggregate (MTA), Cavit, Zinc oxide cement based on mixture of eugenol and ethoxy benzoic acid(Super-EBA), composite resin, amalgam, glass-ionomer(GI) cement and intermediate restorative material [ 4 ]. GI cements are one of the most common restorative materials that are widely used in dentistry. GI ingredients include strontium aluminosilicate glass powder (base), calcium, and a water-soluble polymer (acid) [ 5 ]. GI cements are generated from reaction of weak polymeric acids with powdered glasses. An important clinical advantage of GIs is their adhesion to the surface of the tooth. Adhesion helps the retention of GI cements in the tooth and results in less marginal leakage [ 6 ]. GIs have some properties, including the following. They have a triple cure and setting that improves the quality of polymerization and decreases microleakage. Water sorption in GI restorations can decrease intervals between tooth edges and therefore can show a low microleakage rate in comparison with composite resins [ 7 ]. Use of unfilled resin on the restoration can improve GI properties due to a decrease in its dehydration and results in a decrease in microleakage [ 8 ]. Kolahduzan *et al*. [ 9 ] showed that coronal microleakage with the use of GI was lower than that with other materials, but the differences were not significant.

According to benefits of GI as a restorative material, which is used in sealing of coronal part of root canals in endodontically treated teeth, one of the major issues with the use of GI is determining its optimum thickness. The aim of this study was to compare the effects of different GI thicknesses on microleakage in endodontically treated teeth. 

## Materials and Method

### Preparation of teeth

In this study, 45 single-rooted human teeth were used. The teeth had been extracted for orthodontic or periodontal reasons. The surface of each tooth was cleaned with a Gracey curette. The teeth were stored in 0.9% saline solution at 4°C until used for the purpose of the study. 

All the root canals were prepared by crown-down technique up to #40 master apical file. The root canals were obturated with lateral compaction technique using zinc oxide sealer (Grodab chime GmbH, Germany) and gutta-percha (Gapadent, Germany). The samples were sectioned with a diamond saw (Blade XL 12205, 200rpm, Extec Corp, Enfield, CT, USA) 2mm coronal to the cementoenamel junction. The samples were divided into three groups based on different GI thicknesses. In the group 1 to 3, 1 to 3mm of GI was used as coronal barrier respectively. We used light-cured GI (GG Fuji, Japan) by a dycal applicator (Dentsply, Sirona, USA) filling the coronal part of the root canal from the base which gutta percha was removed to the orifice. Light-curing was carried out for 20 seconds (800mw/ cm2) (LED light curing unit, DEMI, Kerr, Ca, USA). 

The samples were stored in normal saline solution at room temperature for 24hours. Then the tooth apices were coated with sticky wax. After that, except the apex, all the tooth surfaces up to CEJ were coated with two layers of nail varnish. All the teeth were immersed in 2% methylene blue solution for 24 hours. The samples were sectioned sagittally with an automatic cutter (Sruers, Denmark). Finally, dye penetration was assessed under a 40× stereomicroscope (Bel MicroImage Analyzer, Bel Photonics, Monza, Italy). Two independent observers evaluated the teeth and dye penetration was scored.

The scoring was carried out as 0 for no dye penetration, “1” when dye penetration was less than a half of the thickness of light-cured GI, “2” when dye penetration was more than half of the thickness of light-cured GI but did not reach gutta-percha, and “3” when dye penetration reached gutta-percha.

### Statistical Analysis

To compare the means of microleakage in different groups, in cases with normal distribution, if the variance was the same, we employed ANOVA; otherwise, Weltch test was performed. However, in cases in which data were not distributed normally, Kruskal-Wallis test was used. The significance level was set at *p*= 0.05.

## Results

**Figure1:**
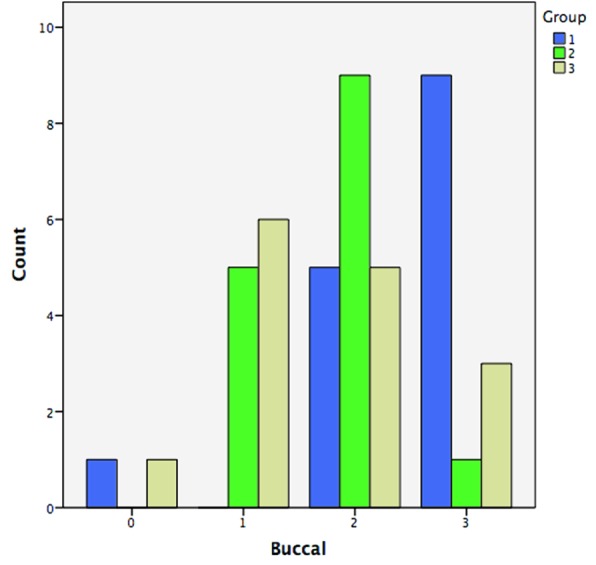
Microleakage score on the buccal surface

**Figure2:**
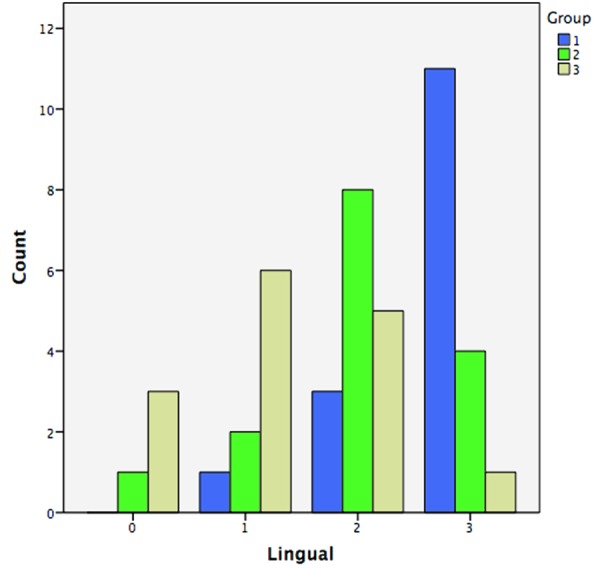
Microleakage score on the lingual surface

**Figure3:**
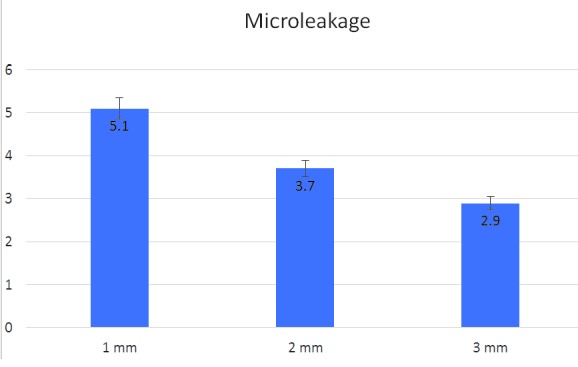
Comparison of coronal microleakage between the groups

Two independent observers evaluated coronal microleakage. First we analyzed the inter-observer agreement. The results showed a kappa coefficient of 0.83% and *p*< 0.001 (Table 1). Given a favorable level of agreement between the observers, we used the reports of observer (#1). The descriptive findings are summarized in Table 2, Table 3 shows the means of dye penetration in groups 1, 2 and 3 were 5.1, 3.7 and 2.9, respectively, with significant differences between the groups. Group 1 exhibited the highest amount of dye penetration while group 3 showed the least (Figures 1 and 2). In addition, comparisons between the groups showed that the means of dye penetration were significantly different between groups 1 and 2 (*p*= 0.002) but the difference was not significant between groups 2 and 3 (*p*= 0.098) (Figure 3). 

**Table1:** Inter-observer agreement

		Value	Asymp. Std. Error	Approx. Tb	Approx. Sig.
Measure of Agreement	Kappa	.836	.070	8.500	.000
No. of Valid Cases		45			

**Table2:** Coronal microleakage on the buccal and lingual surfaces in different groups

Group	Dye penetration score	Lingual	Buccal
Group 1	0	0	1(6.7%)
	1	1(6.7%)	0
	2	3(20%)	5(33.3%)
	3	11(73.3%)	9(60%)
Group 2	0	1(6.7%)	0
	1	2(13.3%)	5(33.3%)
	2	8(53.3%)	9(60%)
	3	4(26.4%)	1(6.7%)
Group 3	0	3(20%)	1(6.7%)
	1	6(40%)	6(40%)
	2	5(33.3%)	5(33.3%)
	3	1(6.7%)	3(20%)

**Table3:** Comparison of dye penetration in different groups

Variable	Mean
Group 1 1mm	5.133333
Group 2 2mm	3.733333
Group 3 3mm	2.933333

Comparison	*p* Value
Group 1& Group 2	*p*= 0.002
Group 1& Group 3	*p*< 0.001
Group 2 & Group 3	*p*= 0.098

## Discussion

GIC have many advantages, including chemomechanical dentin bonding, fluoride release, and thermal expansion like that of tooth structure. GIs are used in the sandwich technique, for coronal sealing and as a coronal barrier during internal bleaching.

Therefore, their important usage is to prevent microleakage [ 10 ]. One of the most important concerns in using GIs is to determine the optimal thickness for effective prevention of microleakage. Therefore, the aim of this study was to evaluate microleakage in three different thicknesses of GI using dye penetration technique.

Our findings showed that the mean dye penetration scores in groups 1, 2 and 3 were 5.1, 3.7 and 2.9, respectively, with significant differences between the groups. Group 1 showed the highest amount of dye penetration while group 3 showed the least. In addition, comparisons between the groups showed the mean dye penetration scores were significantly different between groups 1 and 2 (*p*= 0.002); however, the difference was not significant between groups 2 and 3 (*p*= 0.098). The results not only showed that GIs can effectively prevent coronal microleakage but also the thickness of GI is vitally important for the success of treatment. A thickness of 1mm was the least effective in preventing microleakage; a 2-mm thickness was as effective as the 3-mm thickness in protecting the buccal surface but not the lingual surface. Therefore, to achieve the most effective protective role of GI a 3-mm thickness should be used.

To the best of our knowledge, the present study for the first time compared the effect of different GI thicknesses on coronal microleakage. However, many studies have compared the effect of GI with those of other materials such as composite resin on preventing microleakage. Sherwood *et al*. [ 11 ] showed that at least a 4-mm thickness of GI is needed when 30% H2O2 is used for bleaching. In addition, Diwanji *et al*. [ 12 ] compared microleakage with the use of three different GI products. The teeth were placed in acridine dye and a thermocycler and were sectioned after 24 hours. The highest microleakage was seen with Fuji IX GI, followed by LC II; the lowest microleakage was seen using KN 100 GI. Moreover, Kolahduzan *et al*. [ 9 ] showed that microleakage with the use of GI was lower than that with other materials, but the differences were not significant. Damman *et al*. [ 13 ] evaluated the effect of GI and composite resin, with and without using 1-mm thickness of Coltosol, on microleakage in endodontically treated teeth. In their study, after endodontic treatment 1 mm of gutta-percha was removed from the root canal and replaced with a different sealer. Finally, no sealer was able to fully prevent microleakage but Coltosol + composite resin, composite resin and Coltosol + Vidrion R were significantly more effective in sealing than GI. Our findings were also consistent with those reported by Damman *et al*. [ 13 ] who showed that GI with a thickness of 1mm exhibited low ability to prevent coronal microleakage. Shetty *et al*. [ 14 ] compared the sealing abilities of composite resin, type II GI, amalgam and Ketac Molar in endodontically treated teeth. In this study, 3mm of gutta-percha was removed and replaced with different sealers. They reported GI with a 3-mm thickness resulted in more microleakage compared to composite resin and amalgam but lower than Ketac Molar. In their study, the teeth were immersed in 2% methylene blue to evaluate dye penetration. However, we immersed the teeth for only 24 hours. Therefore, it seems that in the long-time exposure, GI has lower resistance to dye penetration than amalgam and composite resin. Finally, Barekatain *et al*. [ 15 ] compared the sealing ability of two different composite resins and resin-modified glass-ionomer (RMGI) as intra-orifice barriers. They used a 3-mm thickness and showed that microleakage in GI and composite resin was 0.945mm and 0.641mm, respectively, with no statistically significant difference. In that study, the teeth were immersed in methylene blue for 48 hours, twice that in our study. Moreover, Barekatain *et al*. [ 15 ] reported the results quantitatively while we reported them qualitatively. 

## Conclusion

According to the results of the present study, using the higher thickness of GI might decrease coronal microleakage. Overall, our findings indicated that GI in 3-mm thickness showed the highest preventive effect on coronal microleakage in endodontically treated teeth. 
